# Characteristics of clinical trials for non-small cell lung cancer therapeutic vaccines registered on ClinicalTrials.gov

**DOI:** 10.3389/fimmu.2022.936667

**Published:** 2022-10-19

**Authors:** Wenyue Gu, Yangjie Xu, Xiaohong Chen, Hao Jiang

**Affiliations:** ^1^ Department of Pathology, The Yancheng School of Clinical Medicine of Nanjing Medical University, Yancheng Third People's Hospital, Yancheng, China; ^2^ Department of Oncology, Affiliated Cixi Hospital, Wenzhou Medical University, Ningbo, China; ^3^ Intensive Care Unit, The Yancheng School of Clinical Medicine of Nanjing Medical University, Yancheng Third People's Hospital, Yancheng, China; ^4^ Department of Oncology, Zhejiang Hospital, Hangzhou, China

**Keywords:** clinical trials, lung cancer, active immunotherapy, vaccines, cancer therapeutic vaccines

## Abstract

**Background:**

Even after complete surgical treatment or chemotherapy, Non-Small Cell Lung Cancer (NSCLC) patients are also at substantial risk for recurrence and spread trend. Therapeutic cancer vaccination could increase the anti-tumor immune response and prevent tumor relapse. This study aimed to assess the characteristics of NSCLC therapeutic vaccines registered on ClinicalTrials.gov.

**Methods:**

We conducted a cross-sectional, descriptive study of clinical trials for Non-Small Cell Lung Cancer Therapeutic Vaccines Registered on ClinicalTrials.gov (https://clinicaltrials.gov/) through March 17, 2022.

**Results:**

This study encompassed 117 registered trials included for data analysis. The number of trials was significantly correlated with a beginning year (r = 0.504, P < 0.010). Of these trials, 45.30% were completed, 12.82% were terminated, and 8.55% were withdrawn. More than half of trials (52.99%) were funded by industry, and more than half of trials (52.14%) were located in economically developed North America. Regarding study designs of these trials, 27.35% were randomized, 52.14% were single group assignment, 83.76% were without masking, 35.90% were phase 1, and more than half of the trials (56.41%) recruited less than 50 participants. The highest proportion of vaccine types was protein/peptide vaccines (41.88%). Regarding TNM staging, the highest proportion of the trials is stage III-IV (26.50%).

**Conclusion:**

The number of clinical trials about the cancer therapeutic vaccines was sustained an increase in recent years. The main characteristic of clinical trials for NSCLC therapeutic vaccines is lack of randomized control, lack of mask, and recruiting less than 50 participants. In recent years, the protein/peptide vaccines for NSCLC active immunotherapy have been well studied.

## Introduction

Among malignant tumors, lung cancer is the leading cause of death worldwide (1). With the increased deterioration of the environment, the incidence of lung cancer is still on the rise, and its 5-year survival rate is only about 15% (2). Non-small cell lung cancer (NSCLC) accounts for approximately 80% to 85% of all lung cancers (3). Conventional therapies for NSCLC include surgery, radiation, and chemotherapy (4). However, up to 40% of early-stage lung cancer recurred after surgical resection (5). Chemotherapy and radiotherapy are limited by normal-tissue toxicity (6); moreover, tumor cells tend to develop drug resistance in response to chemotherapy (7). Science’s biggest breakthrough in 2013 was immune therapy for cancer by the journal Science (8). In general, tumor immunotherapy involves adoptive cellular immunotherapy, cancer vaccines, immune checkpoint blockade therapy, gene therapy, and immune cell therapy (9, 10). Therapeutic cancer vaccines are based on specific stimulation of the immune system using tumor antigens or tumor cells to elicit an anti-tumor response (9, 11, 12). In recent years, therapeutic cancer vaccines appear to be a very promising strategy for a therapeutic strategy for cancer (13, 14). Notable examples are chimeric antigen receptor T cells and immune checkpoint blockade, providing clinical benefits in different malignancies (15, 16), and leading to their approval by regulatory agencies as well as to the 2018 Nobel Prize in Medicine (17).

Clinical trials are the most effective strategy for evaluating the efficacy of a drug on a specific disease (18, 19) and are a critical step in the successful development of more effective drugs (20). Thus, one of the most important aspects of laying the foundation for future clinical practice is analyzing registered clinical trials. ClinicalTrials.gov is a public trials registry provided by the U.S. National Library of Medicine and the U.S. Food and Drug Administration, accounting for more than 80% of all studies in the World Health Organization’s International Clinical Trials Registry Platform (21). Therefore, to better evaluate the breadth of cancer therapeutic vaccine treatments for NSCLC, we performed a cross-sectional study to investigate the characteristic of registered trials in ClinicalTrials.gov regarding cancer therapeutic vaccines in NSCLC therapy.

## Methods

### Search strategy and selection criteria

Clinical trial data registered on the ClinicalTrials.gov (https://clinicaltrials.gov) websites were collected, and we used the advanced search function with the search term “Lung cancer” for “condition or disease” and the term “vaccine” for “Other terms” on March 17, 2022. All identified clinical trials were assessed to obtain records of all studies. The following information and data were extracted: registered number, title, study type, conditions, interventions, locations, start date, the status of the trial, study results, study samples, participant ages, primary sponsor, location, primary purpose, phases of each trial, allocation, intervention model, masking and intervention. Data were imported into Excel sheets (Microsoft Office Excel 2010, Microsoft Corporation) for further analysis. Exclusion criteria: 1) observation studies; 2) Study Subjects not containing NSCLC; 3) non-human studies (Laboratory Analysis); 4) non-vaccine therapy. All trials were then further subclassified according to their study type. We used descriptive statistics to characterize trial categories. Frequencies and percentages were provided for categorical data.

### Data analysis

Descriptive analyses were used, and primary sponsors were classified as the university, hospital, industry, or other sponsors. If different sites were analyzed in the same region, we were entered into the cumulative calculation for that region. Categorical data are reported as frequencies and percentages, and continuous variables as median and interquartile ranges. We assessed the differences between counts of categorical variables using the Chi-squared test. Ordinary Chi-squared analysis was applied for inspection when n ≥ 40 and T ≥ 5, whereas a calibrated Chi-squared test was employed for inspection when n ≥ 40 and 1 ≤ T < 5. Correlations were analyzed using Spearman correlation. All of the analyses were executed using SPSS 20.0 software. *P*-values < 0.05 were considered to be statistically significant.

## Results

### Screening and included trials

**Figure 1:**
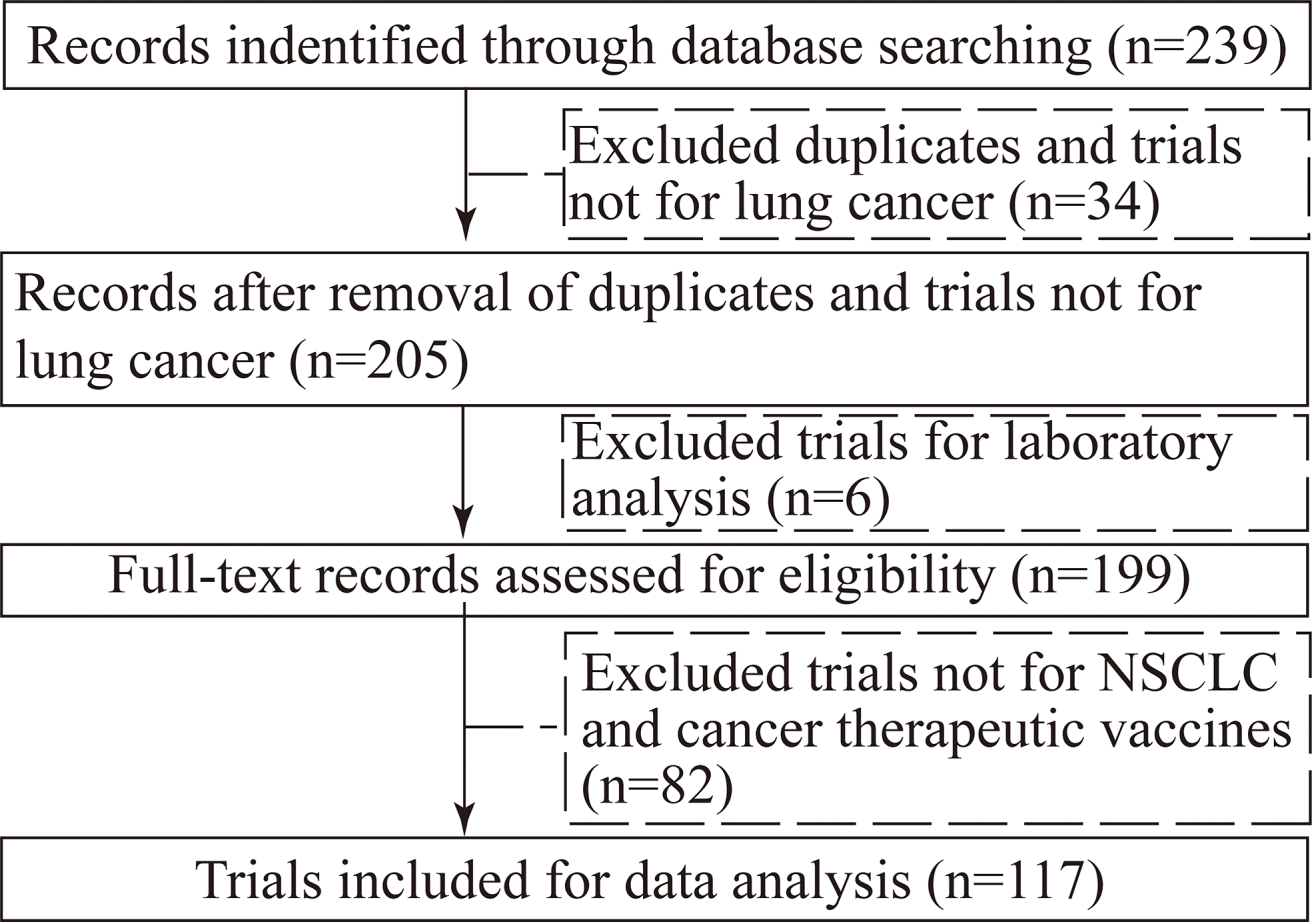
Flowchart of selection trials.

In our initial search, we found 239 clinical trials registered on ClinicalTrials.gov to March 17, 2022; after excluding duplicated trials and trials not for lung cancer, 205 trials remained; after carefully reviewing all the information, 6 trials for laboratory analysis, 60 trials not for NSCLC, 22 trials not for cancer therapeutic vaccines were excluded. Consequently, a total of 117 registered trials were ultimately evaluated (**Figure 1**).

### General characteristics of included trials

**Figure 2:**
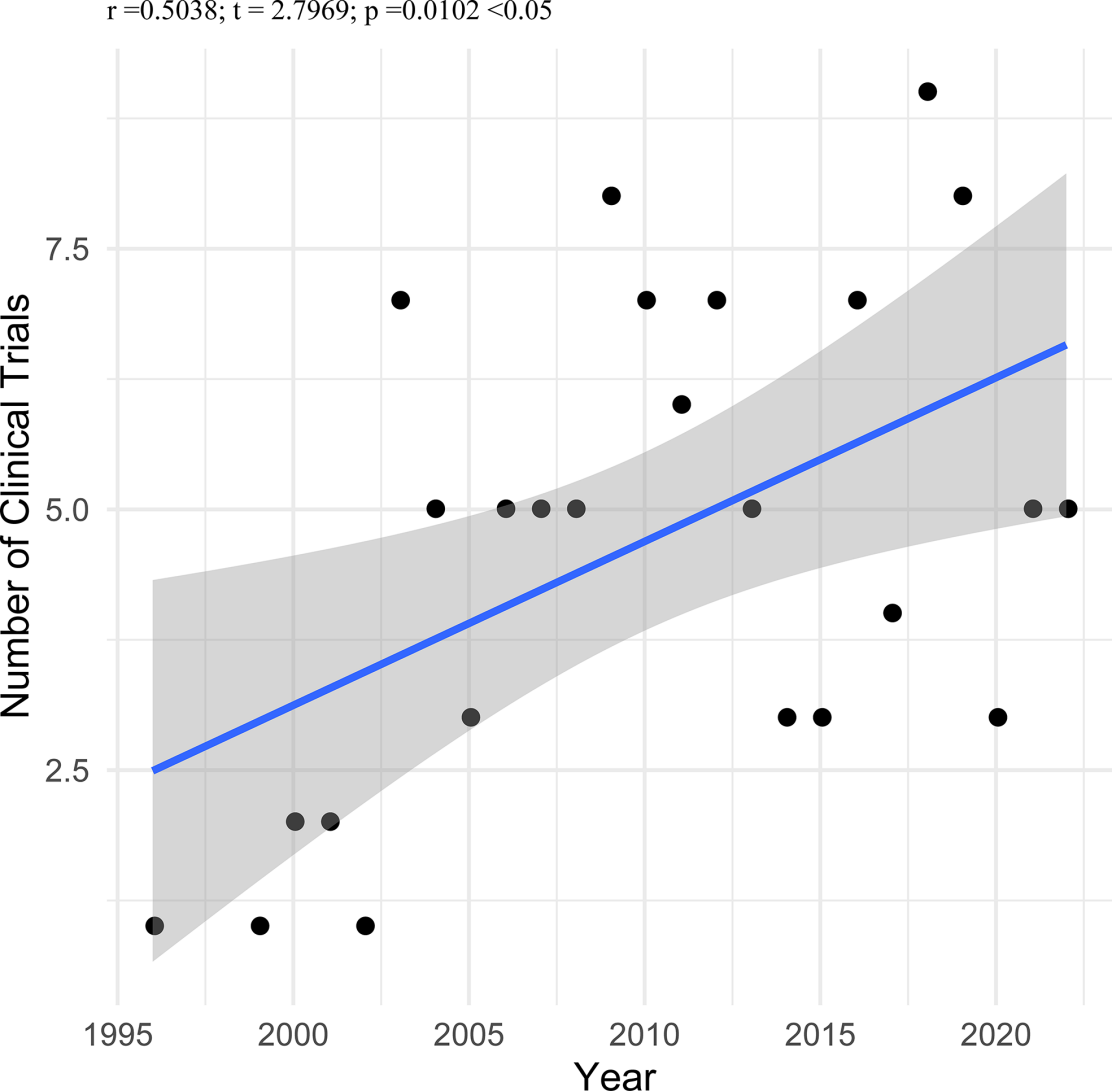
Correlation between the number of trials and beginning year in the 117 included trials.

Only six trials (5.13%) were started before 2002, twenty-one trials (17.95%) between 2002 and 2006, thirty-one trials (26.50%) between 2007 and 2011, twenty-five trials (21.37%) between 2012 and 2016, and there are thirty-four trials (29.06%) since March of 2022. We analyzed the correlation between the number of trials and a beginning year in the 117 included trials. As shown in **Figure 2**, the number of trials was significantly correlated with the beginning year (r = 0.504, P < 0.010). Fifty-three trials (45.30%) were completed, followed by those terminated (12.82%), actively recruited (11.11%), unknown status (11.11%), withdrawn (8.55%), those active but not recruited (5.98%), and those not yet recruiting (5.13%). The majority of trials (85.47%) had no results available; only seventeen trials (14.53%) had resulted on ClinicalTrials.gov. The vast majority of trials (96.58%) recruited adults and older adults as recruited subjects, but four trials (3.42%) selected adults, older adults and child as recruited subjects. More than half of trials (52.99%) were funded by industry, and more than half of trials (52.14%) were located in economically developed North America. The characteristics of included trials are shown in **Table 1**.

**Table 1:** Characteristics of all included trials (n = 117).

Variable	Subgroup	N (%)
Year		
	Prior to 2002	6 (5.13%)
	2002-2006	21 (17.95%)
	2007–2011	31 (26.50%)
	2012–2016	25 (21.37%)
	2017–2022	34 (29.06%)
Status		
	Active, not recruiting	7 (5.98%)
	Not yet recruiting	6 (5.13%)
	Recruiting	13 (11.11%)
	Completed	53 (45.30%)
	Terminated	15 (12.82%)
	Withdrawn	10 (8.55%)
	Unknown status	13 (11.11%)
Study results		
	Has results	17 (14.53%)
	No results available	100 (85.47%)
Age group		
	Adult + Older adult	113 (96.58%)
	Adult + Older adult + Child	4 (3.42%)
Funded by		
	Industry	62 (52.99%)
	NIH	10 (8.55%)
	U.S. Fed	1 (0.85%)
	Industry + NIH	5 (4.27%)
	NIH + U.S. Fed	1 (0.85%)
	Other	38 (32.48%)
Locations		
	Asia	22 (18.80%)
	Europe	32 (27.35%)
	North America	61 (52.14%)
	South America	2 (1.71%)

NIH, National Institutes of Health; U.S. Fed, United States Federal Government.

### Study designs of included trials

The primary purpose of the majority of trials (98.29%) was treatment. The allocation concealment was not clear in 51.28% of these studies. 32 (27.35%) trials were randomized, and 25 (21.37%) were non-randomized. More than half of the intervention models were single group assignments (52.14%), followed by parallel assignments (35.04%), unknown (6.84%), and sequential assignments (5.98%). The majority of trials (98, 83.76%) were without masking, 6 (5.13%) were with unknown masking, and 13 (11.11%) were with masking (2 single maskings, 2 double maskings, 2 triple maskings and 7 quadruple maskings). Trials phases were as follows: early phase 1 (0.85%), phase 1 (35.90%), phase 1/phase 2 (23.08%), phase 2 (26.50%), phase 2/phase 3 (2.56%), phase 3 (6.84%), and not applicable (4.27%). More than half of the trials (56.41%) recruited less than 50 participants, 17 trials (14.53%) recruited 50 – 100 individuals, 13 trials (11.11%) recruited 101 – 500 individuals, 4 trials recruited greater than 500 individuals, and 17 trials (14.53%) did not indicate the number of participants. (detailed data are depicted in **Table 2**).

**Table 2:** Study design elements of included trials (n = 117).

Variable	Subgroup	N (%)
Primary purpose		
	Prevention	1 (0.85%)
	Treatment	115 (98.29%)
	Not applicable	1 (0.85%)
Allocation		
	Randomized	32 (27.35%)
	Non-randomized	25 (21.37%)
	N/A	52 (44.44%)
	Unknown	8 (6.84%)
Intervention model		
	Single group assignment	61 (52.14%)
	Parallel assignment	41 (35.04%)
	Sequential Assignment	7 (5.98%)
	Unknown	8 (6.84%)
Masking		
	Single	2 (1.71%)
	Double	2 (1.71%)
	Triple	2 (1.71%)
	Quadruple	7 (5.98%)
	None (open label)	98 (83.76%)
	Unknown	6 (5.13%)
Phases		
	Early phase 1	1 (0.85%)
	Phase 1	42 (35.90%)
	Phase 1 / Phase 2	27 (23.08%)
	Phase 2	31 (26.50%)
	Phase 2 / Phase 3	3 (2.56%)
	Phase 3	8 (6.84%)
	Not applicable	5 (4.27%)
Enrollment		
	< 50	66 (56.41%)
	50 - 100	17 (14.53%)
	101 - 500	13 (11.11%)
	> 500	4 (3.42%)
	Not applicable	17 (14.53%)

### Overview of investigated vaccine types

**Figure 3:**
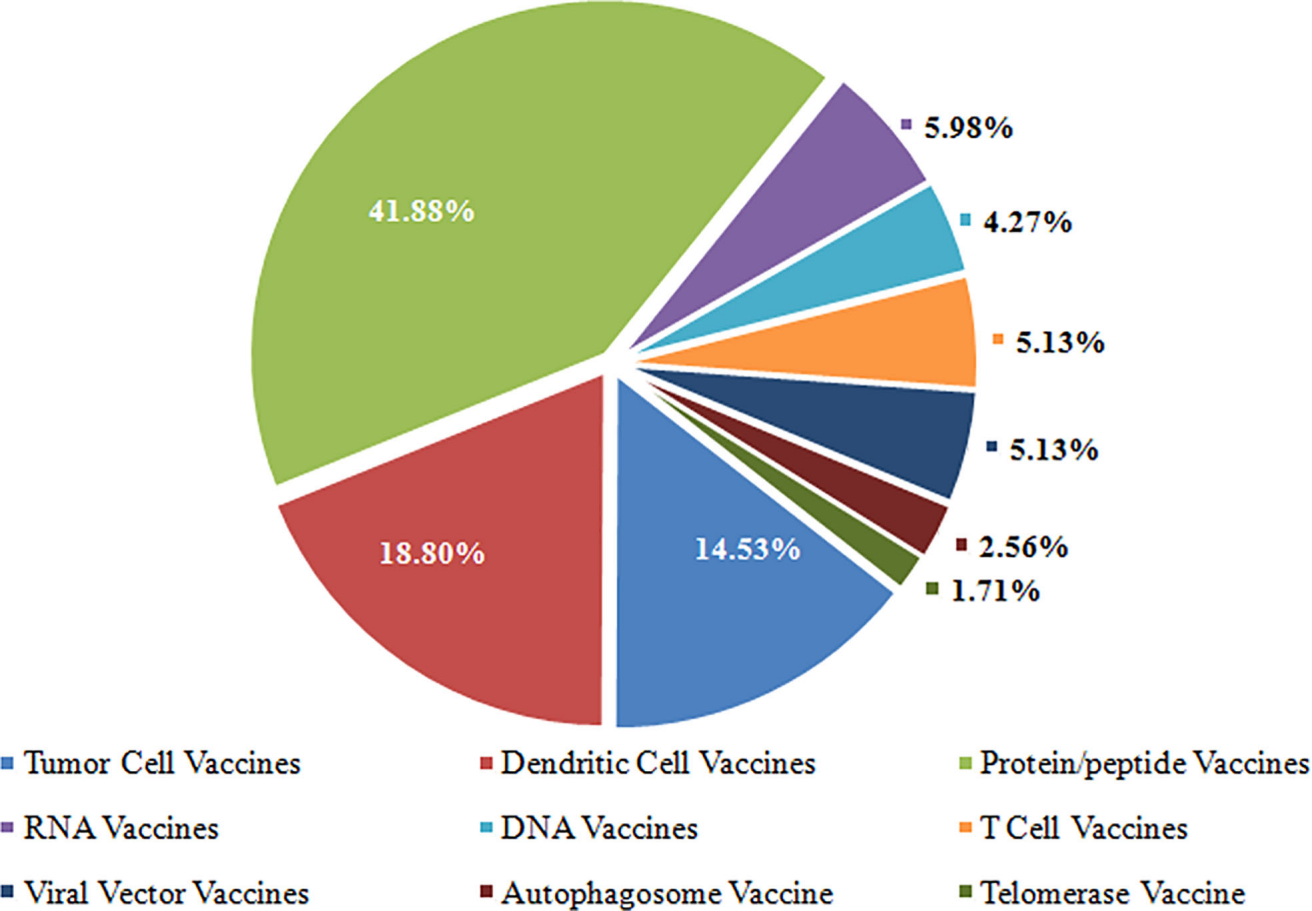
The proportion of various types of therapeutic vaccines.

Cancer cells exploit several mechanisms to evade destruction by the immune system (22), which have led to the development of new tools, including antibodies, peptides, proteins, nucleic acids, and immunocompetent cells (dendritic cells, T cells, etc.) for cancer immunotherapy (23). These techniques fall into seven major categories based on format and content (23, 24), i.e., tumor cell vaccines, T cells vaccines, dendritic cell vaccines, protein/peptide vaccines, DNA vaccines, RNA vaccines, and viral vector vaccines. Of the seven types of cancer therapeutic vaccines, the highest proportion of vaccine types was protein/peptide vaccines (41.88%), followed by dendritic cell vaccines (18.80%) and tumor cell vaccines (14.53%). Detailed data is shown in **Figure 3**.

### Overview of stage of NSCLC and Eastern Cooperative Oncology Group (ECOG) performance status

**Figure 4:**
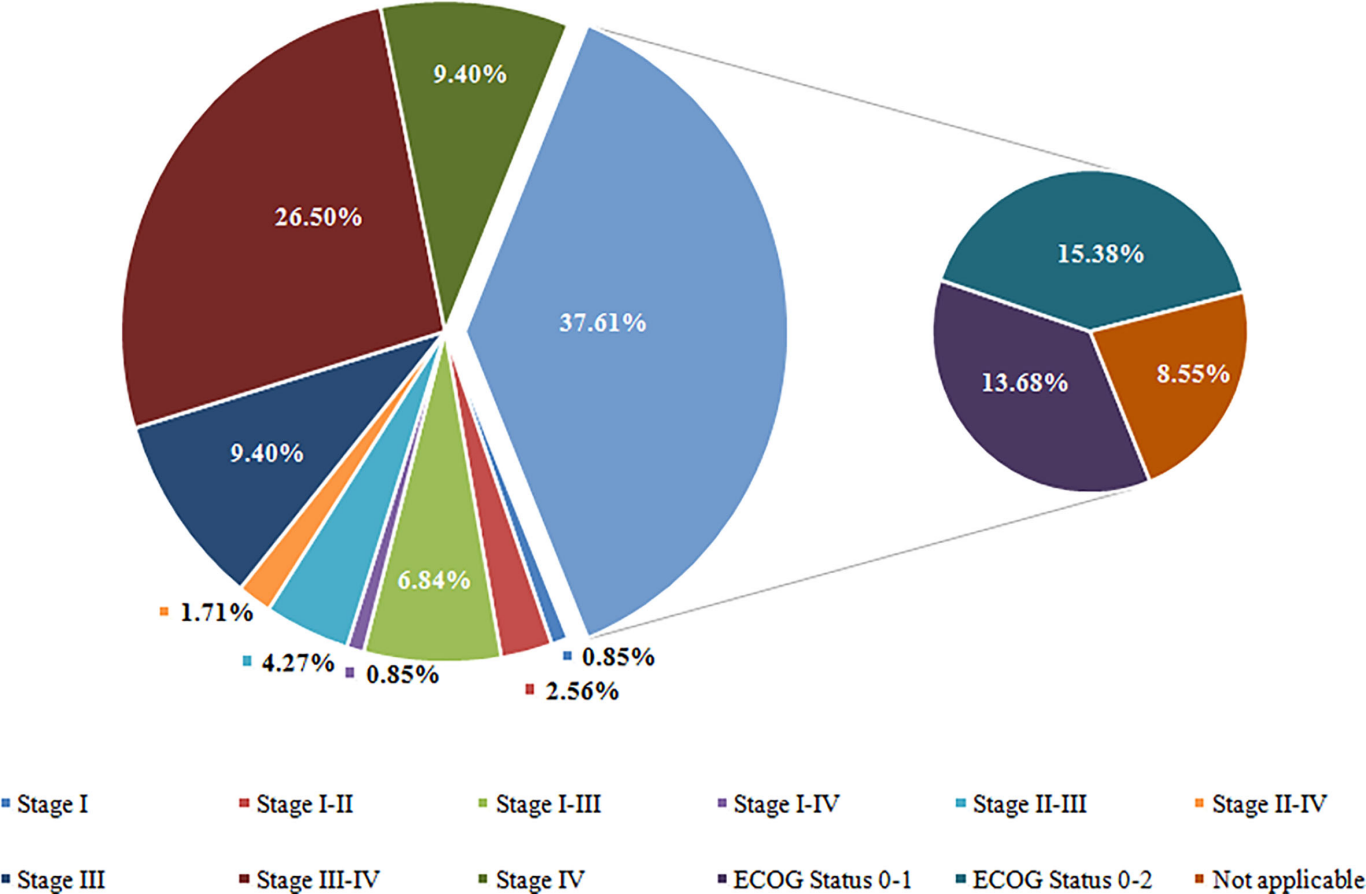
Overview of Tumor Stage for Participate of Included Trials. Stage I including stage I, IA, and IB; Stage II including stage II, IIA, and IIB; Stage III including stage III, IIIA, and IIIB; Stage IV including stage IV, IVA, and IVB. ECOG, Eastern Cooperative Oncology Group.

Of the 117 clinical trials, 73 trials used the Tumor Nodes Metastasis (TNM) staging system as inclusion criteria, and 44 trials used the ECOG performance status as inclusion criteria. Regarding TNM staging, the highest proportion of the trials is stage III-IV (31/117; 26.50%), followed by stage III (11/117; 9.40%), stage IV (11/117; 9.40%), stage I-III (8/117; 6.84%), and stage II-III (4.27%). Eighteen trials (15.38%) recruited NSCLC patients with ECOG status 0 – 2, sixteen recruited NSCLC patients with ECOG status 0 – 1, and ten trials (8.55%) have not applied for TNM staging system or ECOG performance status as inclusion criteria. Detailed data is shown in **Figure 4**.

## Discussion

Even after complete surgical treatment or chemotherapy, patients with NSCLC are at substantial risk for recurrence and spread (25, 26). Therapeutic cancer vaccination, aiming to increase the anti-tumor immune response, overcoming immunosuppression in the tumor microenvironment, could prevent tumor relapse when administered shortly after initial standard treatment when tumor burden is low (27, 28). The cancer therapeutic vaccines have been well studied in recent years (28–30). We analyzed the correlation between the number of trials and the beginning year in the 117 included trials. The number of trials was significantly correlated with the beginning year (r = 0.504, P < 0.010).

There is no uniform consensus on the classification of cancer therapeutic vaccines. Several excellent tools are available to study the mechanisms to avoid immune recognition and destruction by the immune system (23, 24). In this study, the cancer therapeutic vaccines were clustered into several categories according to different origins in these tools, and fall into seven major categories: tumor cell vaccines, T cells vaccines, dendritic cell vaccines, protein/peptide vaccines, DNA vaccines, RNA vaccines, and viral vector vaccines. Of the seven types of cancer therapeutic vaccines, the highest proportion was protein/peptide vaccines (41.88%), followed by dendritic cells (DC) vaccines (18.80%) and tumor cell vaccines (14.53%). With the rapid development of genomic sequencing technologies, cancer-specific antigens in different tumors were identified as cancer-specific cell-surface molecules. Synthesizing molecules with highly specific targets could be a promising avenue for cancer treatment and become a research focus, Such as HLA-A*2402-restricted KIF20A-derived peptide vaccine (31), NEO-PV-01 (32), UV1 (33).

In a review article, Ye et al. detail the various delivery vectors involved in cancer vaccine (30). In this study included trials, DC was the most-used delivery vectors (22/117, 18.80%). DC-based cancer vaccines have been produced, and of the different cancer vaccines available in humans (antigen/adjuvant vaccines, anti-idiotype vaccines, DNA vaccines, tumor cell vaccines) are the most preferred (11).

Clinical trials are critical to clinical practice and decision-making (34). Moreover, Randomized controlled, masked, and appropriate patient-population trials are critical components of high-quality clinical trials (35). In our study, the percentage of randomized trials (27.35%) was lower than in previous studies 84.1% (36); 90.7% (19). More than half of the intervention models were single group assignments (52.14%). 11.11% of trials were with masking, and 56.41% of trials recruited less than 50 participants. The pivotal step in pre-marketing clearance for new drug applications (NDAs) is through filing an application with the Food and Drug Administration (FDA). According to Clinical Trial Endpoints for the Approval of Cancer Drugs and Biologics Guidance for Industry, cancer drugs show effectiveness based on endpoints of tumor assessments, such as disease-free survival, objective response rate, complete response, time to progression and progression-free survival, time to treatment failure (37). Therefore, many cancer drugs come to market-based on single-arm studies, recruiting about 100 participants. Most (60%) of these approvals lack randomized clinical trials during the lifecycle of the product. (38). Randomized clinical trials are the gold standard for shaping clinical practice by providing a definitive evaluation of treatment efficacy (39). So, randomized clinical trials are needed to compare different treatment methods, especially for larger tumors, although we are unaware of any such ongoing trials (40). Bookman et al. believed that international collaboration made randomized trials feasible in rare epithelial ovarian cancer (41). However, it is undeniable that the cancer therapeutic vaccines are an emerging treatment modality for the treatment of cancer, and are currently under evaluation. We found the number of clinical trials of the cancer therapeutic vaccines phase III was lower than in phase II, similar to those reported by Tan et al. in 2015 (42), highlighting a tight bottleneck in drug development processes. Interestingly, more than 50% of NSCLC therapeutic vaccine clinical trials are supported by companies, which implies that cancer therapeutic vaccines have tremendous future potential for transforming the lives of patients with these tumors. Meanwhile, the road to developing cancer therapeutic vaccines for NSCLC is long and expensive, including tissue processing, proteins/peptides identification, vaccine preparation; only big Pharma can support this type of trial. This may also be why most of the trials are focused on protein/peptide vaccines and not on different types of immune system stimulation (dendritic cell, chimeric antigen receptor T-cell, etc.) as it happens in cancers different from NSCLC.

Until now, only one Phase III clinical trial (NCT00409188) for the cancer therapeutic vaccines for NSCLC has been completed, and updated results on ClinicalTrials.gov. The clinical trial is about Cancer Vaccine Stimuvax^®^ (tecemotide formerly known as L-BLP25 or BLP25 Liposome Vaccine) in NSCLC. Tecemotide is an active immunotherapeutic agent targeting a cell surface glycoprotein, mucin 1 (MUC1), and is highly expressed in various malignant tumors and is associated with cellular growth, invasion, and metastasis (43–45). The phase III trial results demonstrated that tecemotide might have a role for patients who initially receive concurrent chemoradiotherapy (46).

There were limitations to the study. We could have missed some clinical trials whose protocol had not been registered in ClinicalTrials.gov, which may register in other country’s clinical trials platforms, such as the International Clinical Trials Registry Platform (https://www.who.int/clinical-trials-registry-platform), the Chinese Clinical Trial Registry Platform (http://www.chictr.org.cn/searchproj.aspx).

## Conclusions

The number of clinic trials about the cancer therapeutic vaccines was sustained an increase in recent years. The main characteristic of clinical trials for NSCLC therapeutic vaccines is lack of randomized control, lack of mask, and recruiting less than 50 participants. In recent years, the protein/peptide vaccines for NSCLC active immunotherapy have been well studied.

## Data availability statement

The original contributions presented in the study are included in the article/supplementary material. Further inquiries can be directed to the corresponding author.

## Author contributions

WG, YX, and HJ planned and drafted the paper. WG and XC contributed to data quality control, analysis, and interpretation. WG, YX, and XC provided administrative guidance and support with data interpretation. YX and HJ led the overall planning and data interpretation. All authors reviewed and revised the manuscript.

## Funding

This study was supported by the Natural Science Foundation of China (82174018), Natural Science Foundation of Zhejiang Province (LY20H280013), Zhejiang Medical and Health Science and Technology Project (2021KY011, 2022KY470), and Zhejiang TCM Science and Technology Project (2022ZB005).

## Conflict of interest

The authors declare that the research was conducted in the absence of any commercial or financial relationships that could be construed as a potential conflict of interest.

## Publisher’s note

All claims expressed in this article are solely those of the authors and do not necessarily represent those of their affiliated organizations, or those of the publisher, the editors and the reviewers. Any product that may be evaluated in this article, or claim that may be made by its manufacturer, is not guaranteed or endorsed by the publisher.
